# Neuropathology of transgenic HD animal models

**DOI:** 10.1186/1750-1326-7-S1-L17

**Published:** 2012-02-07

**Authors:** Xiaojiang Li, Shihua Li

**Affiliations:** 1 Department of Human Genetics, Emory University, Atlanta, GA, USA

## Background

Identification of the polyglutamine expansion in huntingtin (htt) responsible for Huntington disease (HD) has allowed the establishment of a variety of transgenic mouse models of HD. Although these mouse models have been widely used to uncover the pathogenesis of HD and to develop its treatments, most of these mouse models show no overt neurodegeneration in their brains. Similarly, transgenic mouse models for other neurodegenerative diseases such as Alzheimer’s and Parkinson’s diseases do not display obvious apoptosis or significant neurodegeneration either.

## Methods

To address the effect of protein context rather than overexpression or ectopic expression of transgenes, we used the gene-targeting approach to generate a knock-in mouse model that expresses N-terminal mutant htt under the control of the endogenous mouse htt promoter. This mouse model also shows the preferential accumulation of mutant htt in the striatum and age-dependent neurological phenotypes, providing the strong evidence for the pathogenic role of N-terminal mutant htt. To compare the effects of different species on HD neuropathology, we generated transgenic pigs and mice that express the same mutant htt.

## Results

Expression of this N-terminal mutant htt leads to more severe neurological phenotypes in transgenic pigs than transgenic mice. Moreover, mutant htt can cause apoptotic cells in the pig brains, but not in the transgenic mouse brains.

## Conclusion

These studies suggest that species differences and protein context of mutant htt determine the nature of neuropathology in HD and underscore the importance of utilizing different transgenic animal models to understand the pathogenesis of HD.

